# [WITHDRAWN] Hexokinase-1 mitochondrial dissociation and protein O-GlcNAcylation drive heart failure with preserved ejection fraction

**DOI:** 10.21203/rs.3.rs-2448086/v1

**Published:** 2023-01-25

**Authors:** Yuki Tatekoshi, Jason S. Shapiro, Mingyang Liu, George M. Bianco, Ayumi Tatekoshi, Adam De Jesus, Navid Koleini, J. Andrew Wasserstrom, Wolfgang H Dillmann, Samuel E. Weinberg, Hossein Ardehali

**Affiliations:** 1 Feinberg Cardiovascular and Renal Research Institute and Department of Medicine (Cardiology), Northwestern University, Chicago, IL, USA; 2 Department of Medicine, University of California, San Diego, La Jolla, California.; 3 Department of Pathology, Northwestern University Feinberg School of Medicine, Chicago, IL, USA

## Abstract

The authors have requested that this preprint be removed from Research Square.

